# Composite dietary antioxidant index associated with delayed biological aging: a population-based study

**DOI:** 10.18632/aging.205232

**Published:** 2024-01-02

**Authors:** Huiqin He, Xin Chen, Yiming Ding, Xiaoli Chen, Xingkang He

**Affiliations:** 1 Department of Gastroenterology, Sir Run Run Shaw Hospital, Zhejiang University Medical School, Hangzhou 310016, China

**Keywords:** composite dietary antioxidant index, PhenoAge, national health and nutrition examination surveys, aging

## Abstract

Objective: The objective of this study was to explore the potential correlation between the composite dietary antioxidant index (CDAI) and biological aging, addressing the insufficient epidemiological evidence in this area.

Methods: Participants meeting eligibility criteria were selected from the National Health and Nutrition Examination Surveys (NHANES) conducted between 2001 and 2018. CDAI was determined based on dietary antioxidants obtained from 24-hour dietary recalls. Biological age was determined using PhenoAge algorithms incorporating various clinical features. Weighted multiple models were employed to investigate and assess the association between CDAI and biological age.

Results: Analysis of the CDAI quartile revealed disparities in terms of age, gender, ethnicity, educational level, marital status, poverty, dietary calories intakes, smoking, drinking status, BMI, physical activity, and PhenoAge. After adjusting for potential confounding factors, a significant inverse relationship was found between CDAI and Phenotypic Age, with each standard deviation increase in CDAI score correlating with a 0.18-year decrease in Phenotypic Age. These negative correlations between CDAI and PhenoAge advancement were observed regardless of age, gender, physical activity status, smoking status, and body mass index.

Conclusions: Our findings demonstrate a positive relationship between higher CDAI scores and delayed biological aging. These results have significant implications for public health initiatives aimed at promoting healthy aging through dietary interventions.

## INTRODUCTION

As the worldwide population continues to age, aging is set to become a significant health issue. Forecasts suggest that by the year 2030, roughly one-sixth of the global population will be aged 60 years or above [1]. Despite advances in our understanding of aging, there is still much to be learned about the complex processes that underlie age-related diseases [2]. Consequently, it is vital to determine effective strategies for promoting healthy aging. Nonetheless, defining reliable biomarkers for aging poses a challenge. While chronological age is undoubtedly a significant risk factor for aging-related mortality, it is worth noting that individuals of the same chronological age may exhibit varying susceptibilities to such conditions. This suggests that there are differences in their biological aging processes, and it is crucial to differentiate between chronological time and biological aging [3]. Several measures of biological aging have been put forward, encompassing molecular indicators such as telomere length, DNA methylation age, and serum Klotho concentration [4–7]. These have been deemed more dependable predictors of aging outcomes. However, diverse clinical phenotypes or biomarkers, could be employed to assess aging. These measurements might be more pertinent for predicting health outcomes and could offer a more comprehensive assessment of aging outcomes than molecular markers alone. PhenoAge is a biological aging clock that estimates an individual’s biological age based on chronological age and clinical biomarkers, and blood cell parameters [8, 9]. It was developed by Levine, et al. and could effectively identify individuals at higher risk of age-related diseases [10]. PhenoAge provided a more comprehensive measure of an individual’s health and can help identify those who may be at higher risk for age-related diseases.

The aging process is caused by an imbalance of free radicals and antioxidants in the body, leading to oxidative stress that accelerates the aging process by causing damage to cells [11]. The role of diet in regulating oxidative stress is pivotal and can serve as an effective means to combat oxidative stress and mitigate the impact of age-related illnesses [12, 13]. Increasing evidence indicates that consuming antioxidant-rich foods, such as blueberries, pecans, and strawberries, among elderly individuals is linked to a reduced risk of age-related illnesses [14, 15]. The role of antioxidants in shielding biological systems from the toxicity of free radicals by serving as oxidant scavengers has been proposed [16]. However, the effectiveness of antioxidants in improving adverse health consequences remains a debatable topic. The Composite Dietary Antioxidant Index (CDAI) Score is a metric that gauges an individual’s antioxidant profile based on the intake of several dietary antioxidants, including manganese, selenium, zinc, and vitamins A, C, and E [17]. The CDAI was devised to evaluate the comprehensive impact of dietary antioxidants on human health. Studies in the past have demonstrated that individuals with high CDAI scores had a lower probability of developing several types of cancer [17, 18]. Our previous study also demonstrated that higher CDAI was associated with a higher level of serum klotho, which is an important antiaging protein [19]. However, the relationship between CDAI and biological aging has not been thoroughly evaluated yet. The aim of this study was to examine and evaluate the relationship between CDAI and biological aging in the US population, using data from the National Health and Nutrition Examination Survey (NHANES).

## MATERIALS AND METHODS

### Study population

**Figure 1:**
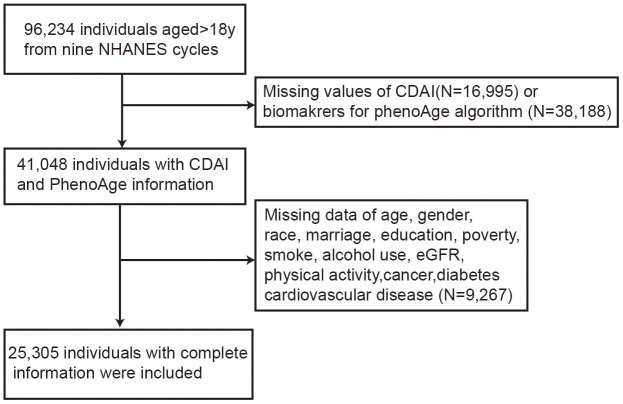
Detailed flowchart for participant selection.

The NHANES program conducted cross-sectional surveys that provided a representation of the non-institutionalized, civilian population of the United States. (https://www.cdc.gov/nchs/nhanes/index.htm). These surveys collected information on demographics, socioeconomic status, dietary habits, and health-related questionnaires through in-person interviews, physical and physiological examinations, and laboratory data. In the current study, nine NHANES cycles (NHANES 2001-2002, 2002-2004, 2005-2006, 2007-2008, 2009-2010, 2011-2012, 2013-2014, 2015-2016, and 2017-2018) were combined for the final analysis. We identified 96, 234 participants aged ≥18 years from nine NHANES cycles and excluded participants with missing information for dietary CDAI score (N=16,995) and biomarkers for PhenoAge calculation (N=38,188). Of the 41,048 individuals with CDAI and PhenoAge information, we excluded participants with missing or incomplete data (N=9,267), resulting in a final sample of 25,305 participants (Figure 1).

### Measurement of CDAI

The Composite Dietary Antioxidant Index (CDAI) estimated the total antioxidant potential of a person’s diet by calculating the sum of the antioxidant scores of various food items, which has been verified in other prospective studies. In summary, it was determined by consuming manganese, selenium, zinc, and vitamins A, C, and E six antioxidants from dietary data. The National Health and Nutrition Examination Survey (NHANES) collected dietary intake information from participants through two 24-hour dietary recall interviews. The first one was done in-person in the Mobile Examination Center (MEC), and the second one was completed by phone between 3 to 10 days later. The United States Department of Agriculture’s Food and Nutrient Database for Dietary Studies was employed to calculate antioxidant, micronutrient, and total energy intakes. Furthermore, questionnaire interviews were conducted to gain insight into the dosage, frequency, and duration of dietary supplement intake during the preceding month.

### Measurement of biological aging

Biological age was measured by the best-validated algorithm that could be implemented with data available in the NHANES, the PhenoAge. Due to its high validity and feasibility of implementation within NHANES, the PhenoAge algorithm was utilized based on clinical laboratory blood chemistries. Briefly, the PhenoAge algorithm was constructed from elastic-net regression on several biomarkers in the NHANES III. This analysis selected following the clinical biomarkers: albumin, creatinine, glucose, white blood cell count, lymphocyte percent, red cell distribution width, mean red cell volume, and alkaline phosphatase. The following formula was used to ascertain the phenotypic age.


$$\begin{array}{l} \text{PhenoAge}=143.5671\\ +\frac{\ln\left[-0.0059383581\times \ln[1-\text{mortality risk}]\right]}{0.08548908}\\ \;\;\;\;\;\;\;\;\;\;\text{mortality risk}=1-e^{-e^{xb_{[\exp(120\text{x}\gamma )-1]/\gamma }}}\\ \;\;\;\;\;\;\;\;\;\;\;\;\;\;\;\;\;\;\;\;\gamma =0.007354285 \end{array}$$

$$\begin{array}{l} xb=-18.311623487-0.029197296\times \text{albumin}\\ \;\;\;\;\;\;+\;0.002285539\times \text{alkaline}\;\text{phosphata}\\ \;\;\;\;\;\;+0.006379140\times \text{creatinine}\\ \;\;\;\;\;\;+0.177752648\\ \;\;\;\;\;\;\times \;\text{glycated}\;\text{hemoglobin}\\ \;\;\;\;\;\;+\;0.055248172\\ \;\;\;\;\;\;\times \;\text{white blood cell count}\\ \;\;\;\;\;\;-\;0.013502137\\ \;\;\;\;\;\;\times \;\text{lymphocyte percentage}\\ \;\;\;\;\;\;+\;0.029331315\\ \;\;\;\;\;\;\times \;\text{mean corpuscular volume}\\ \;\;\;\;\;\;+\;0.234452108\\ \;\;\;\;\;\;\times \;\text{red cell distribution width}\\ \;\;\;\;\;\;+\;0.077245523\times \text{chronological age} \end{array}$$

The BioAge R package was used to implement the PhenoAge algorithm [20]. Owing to the lack of C-reactive protein (CRP) in the NHANES data from 2011 to 2018, we did not include it as a clinical biomarker.

To investigate the effect of CRP on the calculation of PhenoAge, we compared the PhenoAge measurements obtained from a biomarker set without CRP and a biomarker set including CRP and found a strong correlation between them (correlation coefficient was 0.99). In addition, other studies employed clinical biomarkers, without CRP, for the computation of PhenoAge [21, 22]. PhenoAge values indicated the age at which a participant’s mortality risk would match the average in the NHANES III training sample. A higher PhenoAge value implied an accelerated biological aging process, which was associated with an increased risk of age-related diseases and mortality. Conversely, a lower PhenoAge value suggested a slower aging process. PhenoAge advancement was defined as the difference between biological age (PhenoAge value) and chronological age and it was then standardized to have a mean of 0 and a standard deviation (SD) of 1. An increase in PhenoAge advancement value suggested that an individual was experiencing a more advanced state of biological aging (age acceleration), which could increase their risk for diseases and mortality. Conversely, a decrease in PhenoAge advancement indicated a slower rate of biological aging.

### Measurement of covariates

In the current study, we included age, gender (male, female), race (Non-Hispanic white, Non-Hispanic black, Mexican American, others), education level (grade or less, high school, more than high school), marital status (married/living with partner, never married, widowed/divorced/separated), poverty (ratio of family income to poverty), estimating glomerular filtration rate (eGFR), total dietary calories intake (kcal per day), smoking status (never, former, now smoker), drinking status (never, former, mild, middle, heavy), BMI, type of physical activity, history of cancer, cardiovascular disease (CVD), and diabetes as potential covariates in this study. According to the poverty threshold, the ratio of family income to poverty is used to differentiate between low, middle, and high income; those with a ratio of ≤1 are considered low income, those with a ratio of 1 to <4 are considered middle income, and those with a ratio of ≥4 are considered high income. Participants’ smoking status was divided into three categories: current smokers, former smokers, and non-smokers. Those who were considered current smokers smoked on a regular basis and had smoked at least 100 cigarettes in their lifetime. Former smokers had smoked at least 100 cigarettes and had since quit. Non-smokers had either never smoked or smoked fewer than 100 cigarettes. Individuals’ drinking status was classified into five categories: never drinkers (having had less than twelve drinks in their lifetime), former drinkers (having had twelve or more drinks in the past year, but none in the last year, or twelve or more drinks in their lifetime, but none in the last year), current heavy alcohol users (consuming three or more drinks per day for females, four or more drinks per day for males, or engaging in binge drinking), current moderate alcohol users (consuming two or more drinks per day for females, three or more drinks per day for males, or engaging in binge drinking at least twice a month), and current mild alcohol users (consuming one or fewer drinks per day for females, two or fewer drinks per day for males). The average of the data acquired from two 24-hour dietary recall interviews was used to compute the total dietary calorie intake per day. Individuals can be classified into three BMI categories: normal weight (below 25 kg/m^2^), overweight (between 25-30 kg/m^2^), and obese (equal to or above 30 kg/m^2^). Insufficient physical activity was determined to be less than 150 minutes of moderate-intensity exercise per week, while activity was considered to be more than 150 minutes per week. The medical history of the participants concerning cancer, diabetes, and CVD, including conditions such as congestive heart failure, myocardial infarction, coronary heart disease, and stroke, was established using the self-reported information provided by the participants.

### Statistical analysis

We utilized the nhanesR package to search and collect data from the NHANES project. Due to the complex design of this survey, we employed clustering and stratification in our analyses to address unequal selection probabilities and oversampling. Weighted chi-square tests were utilized to compare the characteristics of quintiles of CDAI score for categorical variables, while one-way analysis of variance (ANOVA) was employed for continuous variables. Moreover, a P-value for the trend of difference when CDAI score increased was also determined in Table 1. Several linear regression models were used to evaluate the association between the CDAI score and PhenoAge. Model 1 was adjusted for nothing, while Model 2 was adjusted for age, gender, race, marital status, education level, and family income-to-poverty ratio. Model 3 was further adjusted for smoking, drinking status, eGFR, BMI, total energy intake, physical activity, history of cancer, CVD, and diabetes. We investigated the CDAI score by examining it both as a continuous variable (per one-SD increase) and a categorical variable (moderate (Q2) and high (Q3) versus low (Q1) CDAI score).

**Table 1:** Baseline characteristics of included participants based on quartile of composite dietary antioxidant index (CDAI).

**Characteristic**	**Overall, N = 25305^1^**	**Q1, N = 8436^1^**	**Q2, N = 8437^1^**	**Q3, N = 8432^1^**	**P-Value^2^**	**P for trend**
Age (years)	45.40 (16.06)	45.39 (16.49)	45.91 (16.19)	44.91 (15.54)	0.018	0.98
Gender %					<0.01	
Female	12,143 (48.53%)	4,240 (52.86%)	3,873 (46.47%)	4,030 (46.77%)		<0.01
Male	13,162 (51.47%)	4,196 (47.14%)	4,564 (53.53%)	4,402 (53.23%)		<0.01
Ethnicity %					<0.01	
Non-Hispanic white	12,609 (73.20%)	3,970 (70.38%)	4,341 (74.49%)	4,298 (74.40%)		<0.01
Mexican American	3,827 (7.10%)	1,271 (7.18%)	1,283 (7.06%)	1,273 (7.08%)		0.82
Non-Hispanic black	4,677 (9.01%)	1,838 (11.36%)	1,437 (8.20%)	1,402 (7.77%)		<0.01
Others	4,192 (10.68%)	1,357 (11.08%)	1,376 (10.24%)	1,459 (10.76%)		0.71
Education %					<0.01	
Grade or less	4,991 (12.30%)	2,091 (16.18%)	1,576 (11.71%)	1,324 (9.52%)		<0.01
High school	5,786 (22.97%)	2,167 (27.15%)	1,883 (22.39%)	1,736 (19.93%)		<0.01
More than high school	14,528 (64.73%)	4,178 (56.67%)	4,978 (65.90%)	5,372 (70.54%)		<0.01
Married status %					<0.01	
Married/living with partner	15,817 (65.98%)	4,905 (61.34%)	5,444 (67.84%)	5,468 (68.20%)		<0.01
Never married	4,674 (17.97%)	1,640 (19.49%)	1,461 (16.82%)	1,573 (17.77%)		0.09
Widowed/divorced/separated	4,814 (16.05%)	1,891 (19.18%)	1,532 (15.35%)	1,391 (14.03%)		<0.01
Poverty (ratio of family income to poverty)	3.18 (1.62)	2.87 (1.63)	3.25 (1.59)	3.38 (1.60)	<0.01	<0.01
eGFR (ml/min/1.73m2)	95.47 (20.41)	95.12 (21.27)	95.11 (20.33)	96.13 (19.71)	0.08	
Dietary calories	2,257.11 (1,006.88)	1,571.32 (613.21)	2,217.60 (716.93)	2,884.65 (1,112.51)	<0.01	<0.01
Smoking status %					<0.01	
Never	13,678 (54.40%)	4,235 (49.99%)	4,590 (55.01%)	4,853 (57.61%)		<0.01
Former	6,284 (24.88%)	1,992 (22.57%)	2,189 (25.66%)	2,103 (26.12%)		<0.01
Now	5,343 (20.72%)	2,209 (27.45%)	1,658 (19.32%)	1,476 (16.26%)		<0.01
Drinking status %					<0.01	
Never	3,003 (9.42%)	1,130 (11.10%)	962 (8.95%)	911 (8.41%)		<0.01
Former	3,783 (12.23%)	1,445 (14.34%)	1,252 (11.92%)	1,086 (10.71%)		<0.01
Mild	8,954 (37.75%)	2,683 (32.48%)	3,112 (39.54%)	3,159 (40.59%)		<0.01
Moderate	4,198 (18.36%)	1,354 (18.14%)	1,386 (18.44%)	1,458 (18.48%)		0.72
Heavy	5,367 (22.24%)	1,824 (23.93%)	1,725 (21.15%)	1,818 (21.81%)		0.04
Body Mass Index (kg/m2)	28.52 (6.45)	28.71 (6.59)	28.64 (6.23)	28.25 (6.54)	<0.01	<0.01
Type of physical activity %					<0.01	
Insufficient	7,997 (30.84%)	2,827 (32.90%)	2,657 (30.67%)	2,513 (29.22%)		<0.01
Active	17,308 (69.16%)	5,609 (67.10%)	5,780 (69.33%)	5,919 (70.78%)		<0.01
CDAI	0.99 (4.24)	-2.92 (1.16)	0.11 (0.84)	5.18 (4.15)	<0.01	<0.01
PhenoAge	42.37 (16.93)	42.91 (17.51)	42.76 (16.96)	41.52 (16.34)	<0.01	<0.01
PhenoAge Acceleration	-3.03 (4.62)	-2.48 (4.86)	-3.15 (4.54)	-3.39 (4.45)	<0.01	<0.01
Self-reported chronic diseases						
Cardiovascular diseases^3^ (%)	1,910 (5.9%)	795 (7.4%)	633 (6.0%)	482 (4.4%)	<0.01	<0.01
Diabetes (%)	2,297 (6.8%)	882 (7.8%)	738 (6.4%)	677 (6.3%)	<0.01	0.01
Cancer (%)	2,141 (8.9%)	746 (9.4%)	760 (9.2%)	635 (8.1%)	0.03	0.01

^1^mean (SD) for continuous; n (%) for categorical.

^2^Chi-square tests for categorical variables and one-way analysis of variance (ANOVA) for continuous variables.

^3^Cardiovascular diseases included coronary heart disease, congestive heart failure, stroke and heart attack.

To assess linear trends across the categories of CDAI, we treated the median value of each category as a continuous variable in the models. Multicollinearity among the covariates was assessed using a Variance Inflation Factor (VIF). To evaluate the effect of different variables on the connection between CDAI and biological aging, we conducted a subgroup analysis according to age (<=45 vs >45 years), gender (female vs male), race (Non-Hispanic white vs Non-Hispanic black vs Mexican American vs others), income (low vs middle vs high), physical activity (insufficient vs active), smoking status (never/former vs now), BMI (normal weight vs overweight/obesity) and self-reported chronic diseases (with vs without chronic diseases). Additionally, the P value for the interaction of CDAI score with covariates had been determined. Furthermore, we conducted a sensitivity analysis to address the possibility of reverse causality. Participants with a history of chronic diseases (diabetes, cardiovascular disease, and cancer) that could affect their diet patterns were excluded from the analysis, resulting in a subsample of 10,682 individuals who were free of any of these conditions. Statistical analyses were conducted using R packages, with a significance level set at p<0.05.

### Availability of data and material

The dataset for this study was obtained from NHANES, which is publicly available and can be accessed at the following link: https://www.cdc.gov/nchs/nhanes/.

## RESULTS

The final analysis was conducted on 25,305 participants, who were selected according to the inclusion and exclusion criteria (Figure 1). Table 1 provided a summary of the baseline characteristics of the population stratified by CDAI score. Notable disparities were observed in the CDAI quartiles in terms of age, gender, ethnicity, educational level, marital status, poverty, dietary calorie intakes, smoking, drinking status, BMI, physical activity, and PhenoAge. Individuals who scored higher on the CDAI tended to be younger, male, non-Hispanic Black, more highly educated, married, wealthier, less likely to smoke and drink, have higher energy intake, be more active in physical activity, have lower BMI, and be biologically younger. There was no difference regarding eGFR.

**Figure 2:**
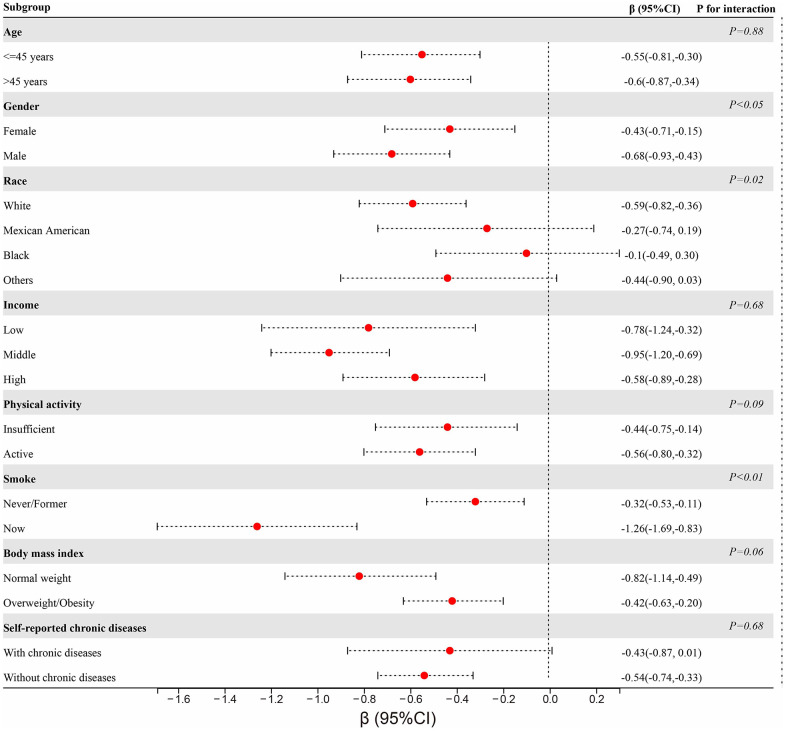
Subgroup analyses of the association between CDAI and PhenoAge stratified by age, gender, race, income, physical activity, smoking, BMI, and self-reported chronic diseases.

After adjusting for multiple covariates, we observed a significant negative association between CDAI and PhenoAge advancement, indicating that individuals with higher CDAI scores had a slower rate of biological aging (Table 2). In the fully adjusted model, each SD increase in CDAI score was associated with a 0.18-year decrease in PhenoAge advancement (Table 2). Participants in the highest quartile of CDAI showed a 0.52-year decrease in PhenoAge advancement as compared to those in the lowest quartile of CDAI (Table 2). The results of the trend test further demonstrated a linear relationship between CDAI score and PhenoAge advancement (Table 2). In the logistic analysis, each SD increase in CDAI score was associated with a lower incidence of accelerated aging. Participants with CDAI scores in the fourth quartile were still significantly associated with a lower incidence of accelerated aging compared with those with scores in the first quartile (Supplementary Table 1). In our subgroup analyses stratified by age, gender, income, physical activity, smoking, BMI, we consistently observed a negative correlation between CDAI and PhenoAge advancement and in all categories (Figure 2). Nevertheless, some disparities were noted among different racial groups and groups with self-declared chronic conditions (Figure 2). A negative correlation was found to be significant only among the white population and those without self-reported chronic diseases; no significant association was observed in other categories (Figure 2). It is noteworthy that, regardless of age, income, and BMI, all subgroups showed statistically significant correlations with the heightened risk of accelerated PhenoAge aging (Supplementary Figure 1). Sensitivity analysis further conducted and results were almost unaltered when participants with chronic diseases, such as diabetes, cardiovascular disease, or cancer, were excluded (Table 3).

**Table 2:** Multivariate linear analysis of the association between composite dietary antioxidant index (CDAI) and PhenoAge advancement.

**CDAI**	**Model I β(95%CI)**	**P**	**Model II β (95%CI)**	**P**	**Model III β (95%CI)**	**P**
Continuous (per SD)	-0.31(-0.40,-0.21)	<0.01	-0.23(-0.32,-0.14)	<0.01	-0.18(-0.27,-0.08)	<0.01
Quartiles						
Quartile 1	Reference		Reference		Reference	
Quartile 2	-0.67(-0.88,-0.47)	<0.01	-0.52(-0.72,-0.33)	<0.01	-0.38(-0.57,-0.19)	<0.01
Quartile 3	-0.91(-1.11,-0.71)	<0.01	-0.66(-0.85,-0.47)	<0.01	-0.52(-0.71,-0.34)	<0.01
P for trend		<0.01		<0.01		<0.01

Model I: non-adjusted model; Model II: adjusted for age gender, race, marital status, education level, and family income-to-poverty ratio; Model III: adjusted for covariates of model 2, and smoking status, drinking status, estimated glomerular filtration rate, body mass index, total energy intake, physical activity, history of cancer, cardiovascular disease, and diabetes.

**Table 3:** Sensitivity analyses of association between composite dietary antioxidant index (CDAI) and PhenoAge excluding participants with chronic diseases.

**Total carotenoid**	**Model I β (95%CI)**	**P**	**Model II β (95%CI)**	**P**	**Model III β (95%CI)**	**P**
Continuous (per SD)	-0.25(-0.35,-0.16)	<0.01	-0.19(-0.29,-0.10)	<0.01	-0.19(-0.29,-0.09)	<0.01
Quartiles						
Quartile 1	Reference		Reference		Reference	
Quartile 2	-0.6(-0.83,-0.37)	<0.01	-0.45(-0.67,-0.22)	<0.01	-0.39(-0.60,-0.17)	<0.01
Quartile 3	-0.8(-1.01,-0.59)	<0.01	-0.59(-0.79,-0.38)	<0.01	-0.54(-0.74,-0.33)	<0.01
Quartile 4	-0.41(-0.47,-0.34)	<0.01	-0.34(-0.40,-0.28)	<0.01	-0.2(-0.26,-0.14)	<0.01
P for trend		<0.01		<0.01		<0.01

Model I: non-adjusted model; Model II: adjusted for age gender, race, marital status, education level, and family income-to-poverty ratio; Model III: adjusted for covariates of model 2, and smoking status, drinking status, estimated glomerular filtration rate, body mass index, total energy intake, physical activity.

## DISCUSSION

In the current study, we revealed that the CDAI had a significant negative impact on the biological age and PhenoAge advancement of the US adult population. These negative associations were consistent across most subgroups, providing additional evidence for the role of antioxidants in anti-aging.

It was a novel finding that CDAI had a noteworthy positive correlation with delayed biological aging. The potential of antioxidants in the diet to protect against age-related diseases, such as cardiovascular disease, certain types of cancers, and neurodegenerative disorders, has been suggested. According to the theory proposed by Harman in 1956, reactive oxygen species (ROC) could be a major cause of aging, causing harm to cells and tissues [23]. As individuals aged, the concentration of oxidized products, including proteins, DNA, and lipids, increased. Antioxidants had been identified as molecules that could reduce reactive oxygen species (ROS) generation and contribute to extending lifespan [24]. Additionally, ROS plays a vital role in the mammalian immune system, serving as a biological defense mechanism that eliminates intracellular pathogens [25]. Therefore, it is widely recognized that an imbalance in redox function can lead to inflammatory reactions. The consumption of antioxidants through the diet is a significant factor that greatly impacts both longevity and the development of aging-related illnesses. The incorporation of exogenous sources of antioxidants is of utmost significance in order to impede the detrimental impact of oxidative stress. These sources include beta-carotene, vitamins C, vitamin E, selenium, zinc, and manganese, as well as Coenzyme Q10 [26]. The depletion of endogenous antioxidants can be arrested through the administration of antioxidant supplements, thereby ultimately mitigating the associated oxidative damage. Plant-derived antioxidants, which are mainly phenolic compounds, vitamins, and flavonoids, can be found in a variety of sources such as fruits, tea, vegetables, nuts, and coffee. These antioxidants have been shown to have a positive impact on various diseases, including cardiovascular disease and diabetes mellitus [27]. It has been observed that a healthy diet is crucial in preventing the harmful effects of oxidative stress and is therefore highly recommended [11]. The CDAI is a frequently employed tool for comprehensively measuring the total antioxidant levels present in a given diet across a variety of research studies. Increasing evidence indicates that CDAI score is related to age-related diseases. Yu et al. investigated the relationship between CDAI score and the likelihood of colorectal cancer (CRC) and found that the lower the CDAI, the higher the risk of CRC [17]. Chen et al. found that CDAI negatively correlated with osteoporosis among middle-aged and older US populations [28]. According to Wang et al., there was a correlation between a high CDAI and a lowered risk of all-cause and cardiovascular mortality. Furthermore, the consumption of antioxidant-rich diets has been shown to significantly prevent the occurrence of cardiovascular mortality [29]. Our previous study also demonstrated that CDAI was significantly associated with serum Klotho levels, an important anti-aging protein in the middle-aged population [19]. In summary, these findings indicate that an elevated CDAI score is linked to a decreased susceptibility to age-related illnesses such as cancer and cardiovascular disease [30]. Furthermore, the consumption of antioxidant-rich diets has been shown to significantly prevent the occurrence of cardiovascular mortality [31]. Our previous study also demonstrated CDAI was significantly associated with serum Klotho levels, an important anti-aging protein in middle-aged population [19]. In summary, these findings indicated that a higher CDAI score was associated with a reduced risk of age-related illnesses such as cancer and cardiovascular disease.

Our findings provided additional evidence that the CDAI score was associated with delayed biological aging. This association remained consistent and significant regardless of other important covariates. Consistent with a previous published article, a higher potential for pro-inflammatory diets was linked to both biological aging and phenotypic age [32]. Kresovich et al. also found that four recommendation-based healthy eating indexes were inversely associated with epigenetic age acceleration [33]. The mechanism connecting CDAI to biological aging has yet to be fully understood. It is plausible that this relationship is multifaceted and warrants additional investigation. The capacity of antioxidants to mitigate oxidative stress and inflammation might be one of the mechanisms [34]. Oxidative stress has the potential to cause cellular damage and may be linked to age-related illnesses. Antioxidants work to counteract reactive oxygen species (ROS) and reduce oxidative stress, potentially slowing down the aging process [35, 36]. Inflammation was also linked to aging and age-related diseases, and antioxidants had been demonstrated to curtail the production of pro-inflammatory cytokines [37]. Moreover, antioxidants had the potential to safeguard telomeres from damage and decelerate the pace of telomere shortening [16]. This was particularly significant because shorter telomeres were linked to cellular malfunction, senescence, and an increased susceptibility to age-related ailments [38–40]. Antioxidants had been found to exert an effect on signaling pathways implicated in the aging process and age-related ailments [41]. Specifically, certain antioxidants had been demonstrated to stimulate sirtuins, a group of proteins that oversee a range of cellular functions associated with aging, such as DNA restoration and metabolic processes [42–44].

To the best of our knowledge, this was the first attempt to analyze the dietary antioxidants potential in terms of biological age. This study had several strengths, including a larger sample size, conducting subgroup analysis, and sensitivity analysis. Our findings provided a more comprehensive understanding of the association between CDAI and biological aging. Our study had some limitations. First, the cross-sectional design of this study only allowed us to assess associations at a single point in time. Therefore, we could not determine causality or temporal relationships between these variables. Future studies using longitudinal designs that track changes in CDAI and biological aging over time are needed to establish causal relationships. Second, while our study included a large sample size, it was limited to participants who met specific inclusion criteria. This may have led to selection bias and limits the generalizability of our findings to other populations. Third, although we adjusted for multiple confounding factors in our analysis, residual confounding may still exist due to unmeasured or unknown variables that could affect the association between CDAI and biological aging.

In conclusion, a high CDAI score was significantly correlated with delayed biological aging among US adults. The results suggested that a significant number of dietary antioxidants might be advantageous in warding off the effects of aging.

## Supplementary Material

Supplementary Figure 1

Supplementary Table 1
