# Supplementary material for: Composite dietary antioxidant index associated with delayed biological aging: a population-based study
Source: Aging (Albany NY). 2024 Jan 2;16(1):15–27. doi: 10.18632/aging.205232 (PMC10817368; doi:10.18632/aging.205232)
Supplement: Supplementary Figure 1 [file aging-16-205232-s001.pdf]

SUPPLEMENTARY FIGURE

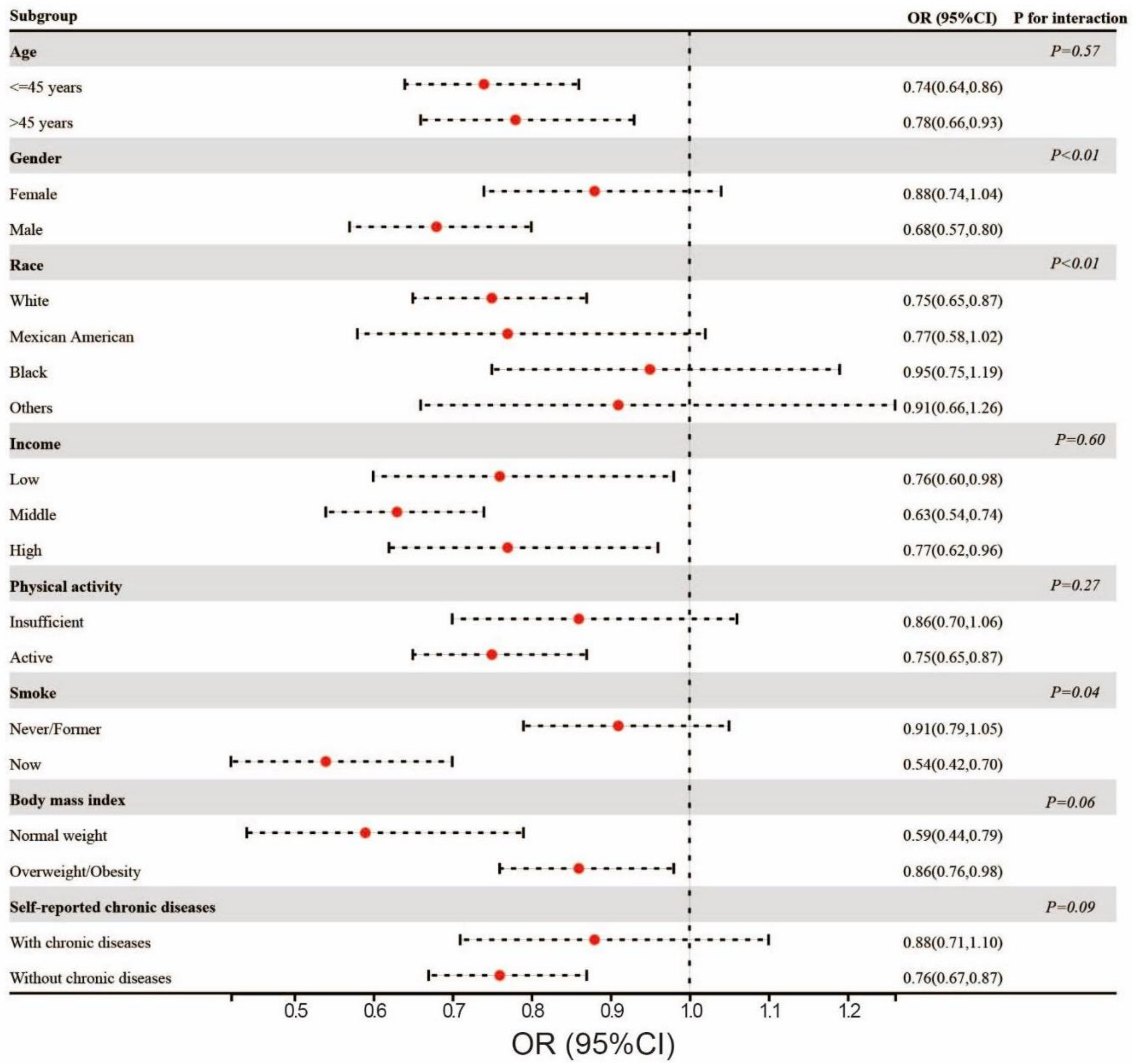

Supplementary Figure 1. Subgroup analyses of the association between CADI and odds ratio of accelerated aging stratified by age, gender, race, income, physical activity, smoking, BMI, and self-reported chronic diseases.
