# Supplementary material for: Composite dietary antioxidant index associated with delayed biological aging: a population-based study
Source: Aging (Albany NY). 2024 Jan 2;16(1):15–27. doi: 10.18632/aging.205232 (PMC10817368; doi:10.18632/aging.205232)
Supplement: Supplementary Table 1 [file aging-16-205232-s002.pdf]

SUPPLEMENTARY TABLE

Supplementary Table 1. Associations of composite dietary antioxidant index (CDAI) with odds ratio of accelerated aging in multivariate logistic analysis.

**Total carotenoid**	**Model I OR(95%CI)**	**P**	**Model II OR(95%CI)**	**P**	**Model III OR(95%CI)**	**P**
Continuous (per SD)	0.87(0.82,0.91)	<0.01	0.91(0.86,0.96)	<0.01	0.91(0.85,0.97)	0.01
Quartiles						
Quartile 1	Reference		Reference		Reference	
Quartile 2	0.73(0.65,0.82)	<0.01	0.79(0.70,0.89)	<0.01	0.81(0.71,0.92)	<0.01
Quartile 3	0.69(0.62,0.76)	<0.01	0.78(0.70,0.86)	<0.01	0.78(0.70,0.87)	<0.01
Quartile 4	0.40(0.35,0.45)	<0.01	0.46(0.40,0.52)	<0.01	0.62(0.54,0.71)	<0.01
P for trend		<0.01		<0.01		<0.01

Model I: non-adjusted model; Model II: adjusted for age gender, race, marital status, education level, and family income-to-poverty ratio; Model III: adjusted for covariates of model 2, and smoking status, drinking status, estimated glomerular filtration rate, body mass index, total energy intake, physical activity, history of cancer, cardiovascular disease, and diabetes.
